# Evaluation of PET Degradation Using Artificial Microbial Consortia

**DOI:** 10.3389/fmicb.2021.778828

**Published:** 2021-12-23

**Authors:** Xinhua Qi, Yuan Ma, Hanchen Chang, Bingzhi Li, Mingzhu Ding, Yingjin Yuan

**Affiliations:** ^1^Key Laboratory of Systems Bioengineering (Ministry of Education), Frontier Science Center for Synthetic Biology, School of Chemical Engineering and Technology, Tianjin University, Tianjin, China; ^2^Collaborative Innovation Center of Chemical Science and Engineering (Tianjin), Tianjin University, Tianjin, China

**Keywords:** artificial microbial consortia, Polyethylene terephthalate, biodegradation, terephthalic acid, ethylene glycol

## Abstract

Polyethylene terephthalate (PET) biodegradation is regarded as an environmentally friendly degradation method. In this study, an artificial microbial consortium composed of *Rhodococcus jostii*, *Pseudomonas putida* and two metabolically engineered *Bacillus subtilis* was constructed to degrade PET. First, a two-species microbial consortium was constructed with two engineered *B. subtilis* that could secrete PET hydrolase (PETase) and monohydroxyethyl terephthalate hydrolase (MHETase), respectively; it could degrade 13.6% (weight loss) of the PET film within 7 days. A three-species microbial consortium was further obtained by adding *R. jostii* to reduce the inhibition caused by terephthalic acid (TPA), a breakdown product of PET. The weight of PET film was reduced by 31.2% within 3 days, achieving about 17.6% improvement compared with the two-species microbial consortium. Finally, *P. putida* was introduced to reduce the inhibition caused by ethylene glycol (EG), another breakdown product of PET, obtaining a four-species microbial consortium. With the four-species consortium, the weight loss of PET film reached 23.2% under ambient temperature. This study constructed and evaluated the artificial microbial consortia in PET degradation, which demonstrated the great potential of artificial microbial consortia in the utilization of complex substrates, providing new insights for biodegradation of complex polymers.

## Introduction

Polyethylene terephthalate (PET) was first used to produce disposable soft bottles in the twentieth century (Koshti et al., 2018). It has been welcomed worldwide and become an indispensable part of people’s lives. However, due to improper treatment strategies and the strong mechanical properties of plastic products, serious environmental problems such as soil pollution and disturbance of marine ecosystems have occurred (Ivar do Sul and Costa, 2014; Yang et al., 2021). Therefore, PET biodegradation has attracted more and more attentions as an environmentally friendly alternative, requiring mild temperature and low energy consumption (Zimmermann and Billig, 2011; Wei and Zimmermann, 2017). The degradation products are easy to be recycled, which is an effective method to control plastic pollution.

Tokiwa and Suzuki (1977) proposed the idea of using enzymes to degrade polymers in 1977. Since then, many PET-degrading enzymes have been discovered and characterized from various microorganisms (Kawai et al., 2019, 2020; Bollinger et al., 2020; Carr et al., 2020; Mohanan et al., 2020; Ballerstedt et al., 2021; Gambarini et al., 2021; Oda et al., 2021). Esterases (Zimmermann and Billig, 2011), cutinases (Ronkvist et al., 2009; Kawai et al., 2014; Sulaiman et al., 2014; Tournier et al., 2020), and lipases (Zhang et al., 2004) have been used in the degradation of PET (Kan et al., 2021). A series of strategies that could enhance the catalytic and activity of PET-degrading enzymes have been proposed (Kawai et al., 2019; Carr et al., 2020; Maurya et al., 2020; Samak et al., 2020; Ballerstedt et al., 2021; Berselli et al., 2021; Gao et al., 2021; Maity et al., 2021; Mohanty et al., 2021).

The mechanism of PET biodegradation is surface hydrophilization of PET films (Kawai et al., 2019). The ends of polymer chains usually protrude, or some polymer chains may form loops, and these are hydrolyzed to carboxylic acid and hydroxyl residues (Kawai et al., 2019). Microorganisms capable of degrading PET first adhere onto the surface of PET films, and then secret PET-degrading enzymes to bind to the substrate (Jaiswal et al., 2020). PET-degrading enzymes mainly act on the ester bond of PET, hydrolyzing it into bis-(2-hydroxyethyl) terephthalate (BHET), monohydroxyethyl terephthalate (MHET), terephthalic acid (TPA) and ethylene glycol (EG). BHET and MHET are both incomplete degradation products, and BHET can be further degraded by PET-degrading enzymes. MHET can be further degraded into TPA and EG under the catalysis of monohydroxyethyl terephthalate hydrolase (MHETase) (Yoshida et al., 2016).

However, most of PET-degrading enzymes like lipases, cutinases and esterases are able to degrade PET under high temperatures (50–70°C), and show very low degradation activity under ambient temperatures (Puspitasari et al., 2020). In 2016, a bacterium named *Ideonella sakaiensis* 201-F6 was isolated from a waste recycling station (Yoshida et al., 2016). It could secret PET hydrolase (PETase) and MHETase to degrade PET into intermediate products at 30°C, providing a basis for biodegradation under ambient temperatures. Since then, the structures of PETase and MHETase have been analyzed, and a series of studies for effective enzyme modifications were carried out, obtaining remarkable results (Han et al., 2017; Austin et al., 2018; Joo et al., 2018; Palm et al., 2019; Ren et al., 2019).

At present, several chassis cells, including *Escherichia coli* (Seo et al., 2019; Shi et al., 2021), *Bacillus* sp. (Huang et al., 2018; Wang et al., 2020), *Pichia pastoris* (Chen et al., 2020), and marine microalgae (Moog et al., 2019; Kim et al., 2020) have been reported to be capable of expressing and secreting PETase. These results established a theoretical basis for the design of PET biodegradation systems. Most studies focused on the initial degradation step. Hydrophobin has been used to convert PET to a hydrophilic form so that it is easier for PETase to contact and thus catalyze the reaction (Ribitsch et al., 2015; Puspitasari et al., 2020). Another study examined how the proximity of the two enzymes influences hydrolytic activity by linking the C terminus of MHETase to the N terminus of PETase (Knott et al., 2020). Purified fusion protein could degrade amorphous PET, and its function was stronger than PETase alone.

PET is a high molecular polymer that cannot enter cells. Enzymatic degradation of PET *in vitro* has been extensively studied, but the purification and preparation process of PETase and MHETase needs additional cost and time of enzyme purification and preparation. PETase and MHETase have relatively higher enzymatic activity at ambient temperature than the other reported enzymes (Yoshida et al., 2016; Papadopoulou et al., 2019; Taniguchi et al., 2019). Considering that the optimal growth temperature of most microorganisms that can produce PET-degrading enzymes is usually 30–40°C, these two enzymes provided a possibility for the direct degradation of PET by microorganisms. In view of the requirements of industrial application, whole cell catalysis is more convenient and practical than enzymes purification. However, the complexity of the metabolic network could increase the physiological burden of chassis cells and restrict their growth.

Additionally, intermediate metabolites are usually toxic. PET breakdown products TPA and EG are both toxic to cells (Gong et al., 2011; Franden et al., 2018). Besides, TPA has been proved to inhibit the secretion of the degrading enzyme (Gong et al., 2011). Accumulation of intermediate metabolites often inhibits the growth of microorganisms and affects degradation efficiency (Knott et al., 2020). The intermediate and final products of PET biodegradation have been identified as competitive inhibitors of PET hydrolases (Vertommen et al., 2005; Barth et al., 2015). To deal with these problems, artificial microbial consortia can be used to mimick natural microbial consortia and perform more complex tasks in a more complex environment (Jones et al., 2017; Krause et al., 2017; Jones and Wang, 2018; Qian et al., 2020). Artificial microbial consortium is artificially designed and synthesized multi-species co-cultured microbial system based on synthetic biology strategies (Ding et al., 2016; Diender et al., 2021). It could achieve the biosynthesis or biodegradation of complex products by integrating the metabolic capabilities of physiologically different species (Ergal et al., 2021). A consortium consisting of three engineered *P. putida*. was constructed to convert PU monomers into rhamnolipids (Utomo et al., 2020). A novel consortium of *Enterobacter* and *Pseudomonas* was constructed to enhance the biodegradation of low-density polyethylene (LDPE) and polypropylene (PP) (Skariyachan et al., 2021). Besides, marine microbial consortia have been used to degrade plasticized unpretreated polyvinyl chloride (PVC) films (containing a total of 30% w/w of additives) by 11.7 ± 0.6% after 7 months (Giacomucci et al., 2020). These results highlight the potential of microbial consortia in plastic degradation.

To explore the great potential of artificial microbial consortia in the utilization of complex polymers, synthetic biology approaches were used to design and construct an artificial microbial consortium for PET degradation, which consisted of two engineered *B. subtilis*, *Rhodococcus jostii* (Rj, Supplementary Table 1) and *Pseudomonas putida* (Pp, Supplementary Table 1). The artificial microbial consortium was proved to efficiently reduce the inhibition of breakdown products TPA and EG, and the degradation efficiency of PET was improved. The strategy of degrading PET by artificial microbial consortia provided novel ideas for the later future degradation of more other types of polymers.

## Materials and Methods

### Materials

PET film (ES301445, amorphous, transparent, 0.25 mm thickness) was purchased from Goodfellow GmbH (London, United Kingdom). BHET (> 85%) was purchased from Tokyo Chemical Industry (Shanghai, China). p-Nitrophenyl acetate (pNPA) was purchased from Sigma-Aldrich (St. Louis, MO, United States). Other chemicals (Supplementary Table 2) used in this study were of analytical grade and purchased from commercial sources.

### Strains, Plasmids, and Primers

*B. subtilis* 168 (*Bacillus* Genetic Stock Center) was used as the starting strain for transformation. *E. coli* Trans1-T1 (TransGene Biotech, Beijing, China) was used for plasmid construction and replication. Wild-type strains *R. jostii* RHA1 (Lindsay Eltis, Univ. British Columbia) and *P. putida* KT2440 (ATCC^®^ 47054) were used for three- and four-species consortia. Microbial strains used in this study are listed in Supplementary Table 1.

### Plasmid Construction and *B. subtilis* Transformation

All plasmids used in this study are listed in Supplementary Table 3. The PETase used in this study was the mutant I179F obtained from previous research (Ma et al., 2018). The PETase gene (GenBank accession number, GAP38373.1) and the MHETase gene (GenBank accession number, GAP38911) were codon-optimized and obtained by GenScript Corporation (Nanjing, China) (Supplementary Table 4). All fragments were amplified using Phanta^®^ Super-Fidelity DNA Polymerase (Vazyme Biotech., Nanjing, China) and were gel purified using a kit (TIANGEN, Beijing, China) before cloning. Fragments of nine common signal peptides were amplified from the *B. subtilis* genome (Supplementary Table 5). The gene PETase was amplified from the synthesized PETase gene (primer pairs shown in Supplementary Table 6), and homologous arms of signal peptides were introduced. The fragments of different signal peptides and PETase were obtained by overlap extension PCR (OE-PCR), digested by *Bam*HI and *Eco*RI, subcloned with linearized pHP13-*P*_43_. This generated plasmids pHP13-*P*_43_-XynA-PETase, pHP13-*P*_43_-BglS-PETase, pHP13-*P*_43_-Csn-PETase, pHP13-*P*_43_-SacB-PETase, pHP13-*P*_43_-PelB-PETase, pHP13-*P*_43_-AmyE-PETase, pHP13-*P*_43_-BglC-PETase, pHP13-*P*_43_-YvpA-PETase and pHP13-*P*_43_-LipB-PETase. Similarly, the fragments of LipB and MHETase were obtained by OE-PCR, generating plasmid pHP13-*P*_43_-LipB-MHETase. Recombinant plasmids were transformed into *B. subtilis 168*, yielding nine strains capable of secreting PETase and one strain that could secrete MHETase. The strains containing recombinant plasmid pHP13-*P*_43_-LipB-PETase and pHP13-*P*_43_-LipB-MHETase were named *B*_*S*__PETase and *B*_*S*__MHETase (B1 and B2, Supplementary Table 1). *B. subtilis* transformation was performed using the two-step method of nutrient degradation (Spizizen, 1958).

### Pretreatment and Culture Condition

#### Pretreatment of Substrate

BHET was dissolved with 50% γ-cyclodextrin solution, and then filtered and sterilized. The degradation reaction of BHET was detected with high-performance liquid chromatography (HPLC). PET film was first cut into 1.0 cm × 1.5 cm sheets and was weighed. Sheets were washed for 1 h each with 1% sodium dodecyl sulfate (SDS), 70% ethanol, then distilled water. PET film was irradiated with UV light for 24 h and added to the medium as a substrate. When degradation reaction was terminated, PET films were removed from the reaction solution, washed with 70% ethanol, dried at 70°C for 30 min, then analyzed with the gravimetric weight loss method.

#### Culture Condition

For initial growth, all bacteria were cultured in 5 mL Luria-Bertani broth (LB) for 12 h with shaking at 220 rpm. *B. subtilis* and *P. putida* were grown at 37°C and *R. jostii* was grown at 30°C. LB for *B. subtilis* additionally contained 2 μg/mL erythromycin.

For PET degradation experiments by PETase, 10 μg of purified PETase (Ma et al., 2018) was incubated with PET film at 30°C for 48 h. The amount of adding exogenous PET monomers was 0.3 mM TPA, 0.6 mM EG, and a mixture of 0.3 mM TPA + 0.6 mM EG.

For PET degradation experiments by the two- or three-species microbial consortium, the initial volume of LB medium was 20 mL. During the 7 days of the degradation process, 10 mL of fresh LB medium was supplemented on the 2nd, 3rd, and 4th days. For PET degradation experiments by the four-species microbial consortium, *B*_*S*__PETase, *B*_*S*__MHETase, *R. jostii* and *P. putida* were cultured overnight in 50 mL of LB medium. Cells were obtained by centrifuging 5 mL of culture medium at 4,000 rpm for 5 min. The bacteria were washed with fresh W_*n*_ medium (Supplementary Table 2) and diluted to OD_600_ = 2. *P. putida* was diluted 1, 10, 10^2^, 10^3^, 10^5^, 10^6^ times, then 100 μL was added to microbial consortia. Bacteria strains, *B*_*S*__PETase, *B*_*S*__MHETase and *R. jostii* were added without dilution (0.3, 0.1, and 1.0 mL, respectively). The final ratio of *B*_*S*__PETase, *B*_*S*__MHETase, *R. jostii* and *P. putida* was 3:1:10:10^–5^, which can be changed by changing the inoculation volume. The consortium was inoculated into 20 mL W_*n*_ medium with 2 μg/mL erythromycin, and 10 mL W_*n*_ medium was supplemented on the 2nd, 3rd, and 4th days of degradation. Each experiment was repeated in triplicate.

#### Terephthalic Acid, Bis-(2-Hydroxyethyl) Terephthalate, and Ethylene Glycol Quantification

After cultivation of consortia with BHET or PET film, samples and standard solutions were purified with a filter membrane to detect the content with high-performance liquid chromatography (HPLC). TPA and BHET were analyzed by using HPLC with a UV detector (Waters, Milford, MA, United States) equipped with a Hypersil OD-2 C18 column (4.6 mm × 250 mm, 5 μm, Thermo Fisher Scientific, Waltham, MA, United States). The mobile phase comprised 40% methanol and 60% formic acid (0.1%, v/v) solution (10 μL injection, 0.6 mL/min, 30°C). The maximum absorption wavelength was 240 nm.

EG was analyzed by HPLC with a refractive index detector (Waters) equipped with an HPX-87H column (7.8 mm × 300 mm; Bio-Rad, Hercules, CA, United States). Sulfuric acid (5 mM) was employed as the mobile phase (10 μL injection, 0.5 mL/min, 60°C).

#### Screening of Signal Peptide for PET Hydrolase Secretion

The engineered *B. subtilis* with different signal peptides (Supplementary Table 5) were grown in 50 mL of LB with 2 μg/mL erythromycin at 37°C with 220 rpm shaking for 24 h. The culture was diluted in 50 mL of fresh LB medium to an initial OD_600_ of 0.2 for shake flask fermentation for 12 h. The activity of PETase was determined using pNPA as substrate. The pNPA was first dissolved in methanol and then diluted with phosphate buffer (50 mM Na_2_HPO_4_-HCl, pH 7.0) to a final concentration of 1 mM. The reaction was triggered by adding 200 μL of substrate solution to the 50 mL of medium. The mixture was incubated at 37°C for 5 min, and the product, p-Nitrophenyl (pNP), was continuously measured at a wavelength of 405 nm.

### Scanning Electron Microscopy

The PET films were washed as the method in section “Pretreatment of Substrate,” and then observed with a scanning electron microscope (SEM). The relevant places in the samples were scanned using an electron microscope at 50,000 × magnification.

## Results

### Construction of a Two-Species Microbial Consortium to Degrade Polyethylene Terephthalate

**FIGURE 1:**
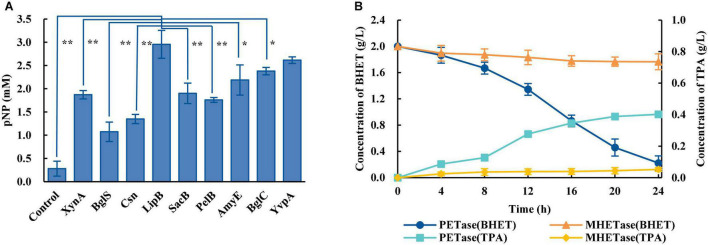
Construction of a two-species microbial consortium for BHET degradation. **(A)** Fusion of the PETase gene to different signal peptides resulted in different levels of PETase secretion, and therefore different levels of the product pNP. The species in the control group were wild-type *B. subtilis*. **(B)** Comparison of the effects of PETase and MHETase on BHET. All experiments were performed at least in triplicate. Error bars indicate standard deviation. *p < 0.05, **p < 0.01 (one-sided *t*-test).

Considering PET is a polymer which is difficult to enter cells, the biodegradation of PET requires microorganisms that can efficiently secrete PET-degrading enzymes. The biodegradation of PET at ambient temperatures includes a two-step reaction: PET is degraded into MHET catalyzed by PETase, and MHET is degraded into TPA and EG catalyzed by MHETase. An engineered *B. subtilis* that can efficiently secrete PETase (*Bs*_PETase, Supplementary Table 1) was first constructed. The screening of a better signal peptide for PETase expression in *B. subtilis* could be carried out, and nine commonly used signal peptides (Supplementary Table 5) were tested. Results showed great differences in secretion of the target protein using the different signal peptides. Signal peptide LipB from extracellular esterase had the best performance, producing 2.98 mM of pNP (Figure 1A). The SEM image of PET film surface cultured with *Bs*_PETase showed obvious structural changes compared with the control group (Supplementary Figure 1). Then, another engineered *B. subtilis* was constructed to efficiently secrete MHETase (*Bs*_MHETase, Supplementary Table 1).

**FIGURE 2:**
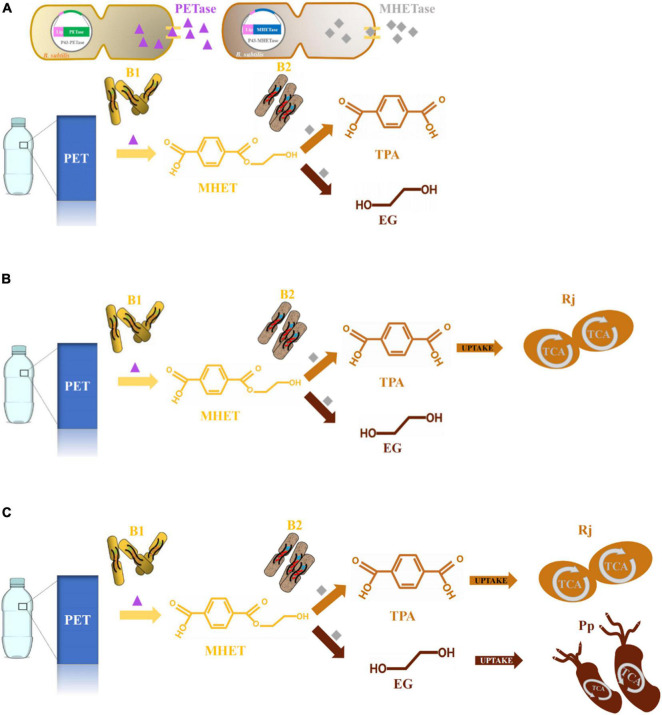
Schematic diagram of PET degradation by synthetic microbial consortia. **(A)** *Bs*_PETase (B1) and *Bs*_MHETase (B2) were engineered to secrete PETase and MHETase to degrade PET and a two-species microbial consortium was constructed. **(B)** Due to PETase inhibition by TPA, *R. jostii* (Rj) was added, resulting in a three-species microbial consortium. **(C)** To further utilize EG, *P. putida* (Pp) was introduced and a four-species microbial consortium was constructed.

BHET was used as the substrate to explore optimal PET degradation. The time required for complete degradation of BHET increased as the concentration of BHET increased. The final BHET concentration used was 2 g/L because it was difficult to completely dissolve in γ-cyclodextrin solution at higher concentrations (Supplementary Figure 2). As shown in the degradation curve of BHET and the generation curve of TPA, PETase showed efficient degradation activity for BHET whereas MHETase did not (Figure 1B). Besides, previous studies additionally showed that PETase has a small amount of activity on MHET, generating TPA and EG (Yoshida et al., 2016). Thus, a two-species microbial consortium composed of two engineered *B. subtilis* (*Bs*_PETase and *Bs*_MHETase, Supplementary Table 1) was designed for degradation of BHET (Figure 2A).

**FIGURE 3:**
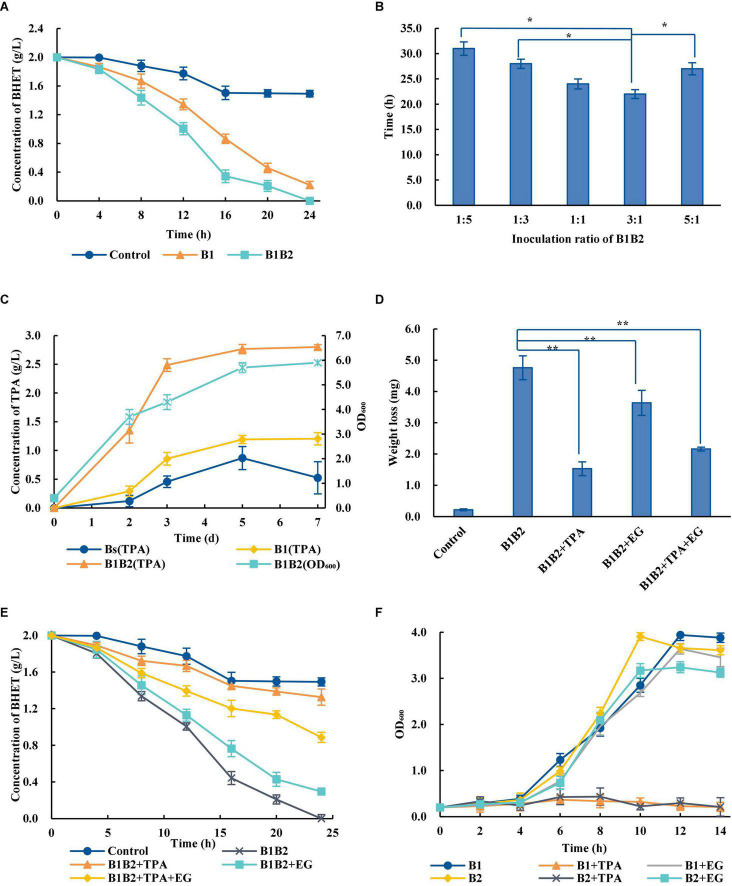
Optimization of the two-species microbial consortium. **(A)** Comparison of BHET degradation by single bacterial species and the two-species microbial consortium. B1, *Bs*_PETase; B2, *Bs*_MHETase. The control group the BHET degradation by wild-type *B. subtilis*. **(B)** Time required for the two-species microbial consortium to completely degrade 2 g/L BHET with different ratios of bacteria added at inoculation. **(C)** Concentration of TPA over time during degradation of PET. **(D)** Effects of adding TPA, EG and TPA + EG on PET weight loss. The addition of exogenous degradation products significantly inhibited the degradation of PET compared with B1B2 (only using *Bs*_PETase and *Bs*_MHETase). **(E)** Effects of adding TPA, EG, or TPA + EG on BHET degradation. **(F)** Effects of adding TPA and EG on *Bs*_PETase (B1) and *Bs*_MHETase (B2). All experiments were performed at least in triplicate. Error bars indicate standard deviation. *p < 0.05, **p < 0.01 (one-sided *t*-test).

The artificial microbial consortia are designed for the complex metabolic network to achieve the optimal process allocation and reduce the cell pressure (Kong et al., 2018). Compared with *Bs*_PETase alone, the degradation efficiency of two-species microbial consortium was significantly improved—2 g/L BHET could be completely degraded within 24 h (Figure 3A). However, wild-type *B. subtilis* also had weak degradation ability, which may be related to its complex metabolic network *in vivo* (Jones and Wang, 2018).

Temperature, pH, and inoculation rate were optimized. Results showed that the changes of temperatures and pH would not affect the degradation rates of PET dramatically (Supplementary Figures 3A,B). Although the optimal growth temperature for *B. subtilis* was 37°C, the optimal temperature for the two-species microbial consortium was 38°C (Supplementary Figure 3A). Different inoculation ratios greatly affected degradation rate (Figure 3B). The optimal ratio of *Bs*_PETase and *Bs*_MHETase at inoculation was 3:1. After optimization, 2 g/L of BHET could be completely degraded within 22 h (Figure 3B), which was significantly improved compared to that before (24 h, Figure 3A). It is likely that increasing the initial amount of *Bs*_PETase increased the amount of PETase, thus improving the degradation effect.

### Effect of Terephthalic Acid and Ethylene Glycol on Degradation Process

A series of optimizations on the two-species microbial consortium was carried out to obtain an appropriate degradation condition. The two-species microbial consortium was used to degrade amorphous PET film. Results showed that the addition of fresh medium could maintain the growth of bacteria (Figure 3C). However, the production of TPA increased rapidly in the first 3 days, then slowed down, and basically reached equilibrium after the fifth day (Figure 3C). It is hypothesized that a byproduct of the reaction may have slowed the process over time. Exogenous addition of EG, TPA and their mixture markedly affected the degradation rates of PET (Figure 3D). The weight of PET film in the group with no PET monomers added decreased by 4.76 ± 0.38 mg (mean ± standard deviation) but only by 1.53 ± 0.22 mg in the group with TPA added (Figure 3D). Although TPA had the highest inhibitory effect on the degradation process, an inhibitory effect of EG was also observed. TPA, EG and the TPA/EG combination also inhibited degradation of BHET (Figure 3E).

In order to further explore the influence why this inhibition occurred, the effect of TPA and EG on bacterial consortium growth was measured. The growth rate of bacteria was severely inhibited in the presence of TPA, whereas the addition of EG had little effect on it (Figure 3F). Therefore, it is speculated that the addition of TPA to the two-species microbial consortium during PET degradation negatively affected bacterial growth, in turn decreasing enzyme production.

### Construction of a Three-Species Microbial Consortium for Polyethylene Terephthalate Degradation

**FIGURE 4:**
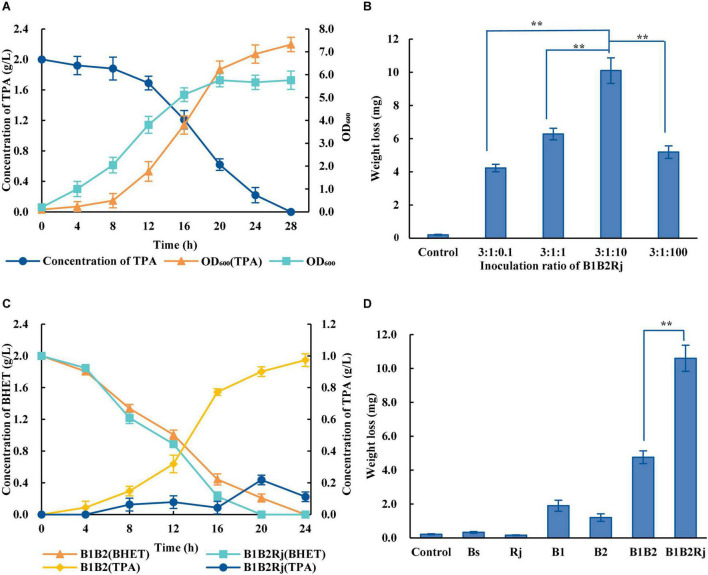
Construction of the three-species microbial consortium. **(A)** Concentration of TPA during incubation with *R. jostii* (dark blue circles) and OD_600_ of *R. jostii* grown in LB medium with (orange triangles) or without (light blue squares) 2 g/L TPA. **(B)** PET degradation by the three-species microbial consortium with different starting ratios of *Bs*_PETase: *Bs*_MHETase: *R. jostii*. **(C)** Concentrations of BHET (orange triangles and light blue squares) and TPA (yellow rhombuses and dark blue circles) over time when incubated with different microbial consortia. **(D)** PET degradation by *Bs*_PETase, *Bs*_MHETase, the two-species microbial consortium, and the three-species microbial consortium. All experiments were performed at least in triplicate. Error bars indicate standard deviation. **p < 0.01 (one-sided *t*-test).

In order to eliminate the inhibitory effect, further degradation of TPA is needed. *R. jostii* RHA1, which was isolated from soil, has extraordinary abilities to degrade a variety of aromatic compounds (Hara et al., 2007; Xiong et al., 2012). *R. jostii* RHA1 was verified to degrade TPA efficiently, with 2 g/L TPA being completely degraded within 28 h (Figure 4A).

A three-species microbial consortium consisting of *B*_*S*__PETase, *B*_*S*__MHETase and *R. jostii* was constructed for BHET and PET degradation to reduce the inhibition of TPA on PETase (Figure 2B). Temperature, pH, and inoculation ratio were optimized. Results showed that different temperatures and pH had almost no effect on degradation rates (Supplementary Figures 3C,D), while the initial concentration of *R. jostii* compared to the other two species affected the degradation rates dramatically (Figure 4B). The optimal inoculation ratio of *Bs*_PETase: *Bs*_MHETase: *R. jostii* was 3:1:10 (Figure 4B). When the three-species microbial consortium was applied to degradation of BHET, there was a relatively low concentration of TPA compared to the two-species microbial consortium, and the inhibitory effect of TPA on PETase was weak; 2 g/L of BHET could be completely degraded within 20 h (Figure 4C). Degradation of PET was also improved with the three-species microbial consortium compared to the two-species microbial consortium. After 7 days, 31.2 ± 2.2% of PET was degraded by the three-species microbial consortium (Figure 4D), compared to 13.6 ± 1.1% with the two-species microbial consortium, achieving an improvement of about 17.6% due to the reduction of inhibitory TPA.

### Construction of a Four-Species Microbial Consortium for Polyethylene Terephthalate Degradation

Like TPA, EG is also a toxic compound with a significant inhibitory effect on the degradation process of PET (Figure 3D), and therefore further degradation strategy for truly safe, green disposal of PET is needed (Devillers et al., 2002). EG can be metabolized into acetaldehyde by *P. putida* KT2440 (Mueckschel et al., 2012; Franden et al., 2018; Li W.-J. et al., 2019). After preliminarily verifying the ability of *P. putida* KT2440 to completely degrade 2 g/L of EG within 20 h (Supplementary Figure 4A), it was introduced into the three-species microbial consortium to generate a four-species consortium. In this consortium, *Bs_*PETase secreted PETase to degrade PET into MHET; *Bs_*MHETase secreted MHETase to degrade MHET into TPA and EG, which were then degraded by *R. jostii* and *P. putida*, respectively (Figure 2C). This consortium therefore effectively both reduced the inhibition effect of TPA and EG and avoided further environmental pollution.

In co-cultivation, *P. putida* strongly inhibited growth of the other three bacteria in nutrient-rich media such as LB, Yeast Extract Peptone Dextrose Medium (YPD), and Synthetic Complete Medium (SC) (Supplementary Table 2); when all four species were cultured in these media for 24 h then the culture was spread on solid growth medium, only *P. putida* was found. It was therefore necessary to identify a suitable medium to prevent the rapid growth of *P. putida* compared to the other species. Inorganic salt media (Supplementary Table 2) have relatively poor nutrition and were chosen for comparison. Among them, W medium had the strongest inhibitory effect on the growth of *P. putida*, allowing it to grow to an OD_600_ of only 2.4 over 24 h. In contrast, *R. jostii* reached an OD_600_ of only 4.6 after 24 h, becoming the dominant strain, and *B. subtilis* reached an OD_600_ of 0.8, nearly 4 times higher than that in the other media tested (Supplementary Figure 4B). The growth of *B. subtilis* was significantly improved with sucrose and glucose as dual carbon sources, and the addition of ammonium sulfate and potassium nitrate (Supplementary Figures 4C,D). BHET (2 g/L) was completely degraded within 20 h by the four-species microbial consortium, TPA and EG were not accumulated (Supplementary Figure 4E).

**FIGURE 5:**
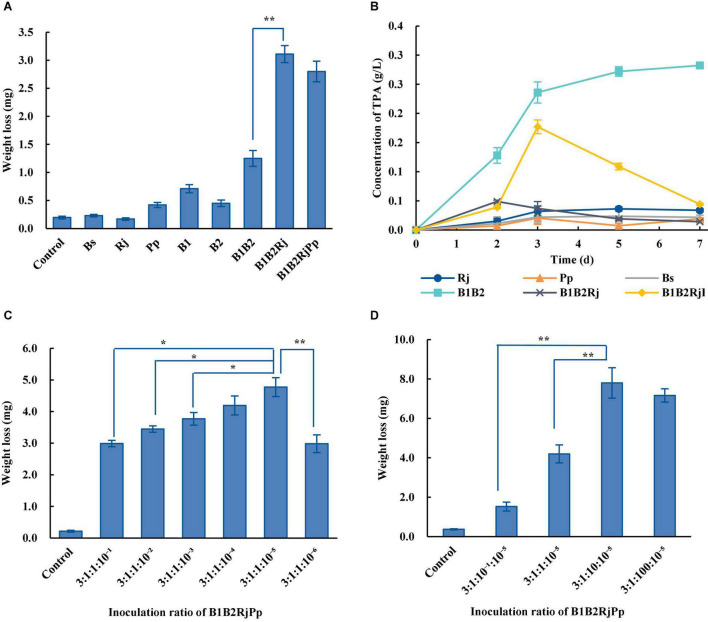
PET degradation by four-species microbial consortia. **(A)** Weight loss (degradation) of PET film incubated with different consortia in Wn medium. **(B)** Concentration of TPA incubated with different consortia in Wn medium. **(C)** Optimization of the initial ratio of *Bs*_PETase/*Bs*_MHETase/*R. jostii to P. putida* in the four-species microbial consortium in Wn medium. **(D)** Optimization of the initial ratio of *Bs*_PETase/*Bs*_MHETase/*P. putida* to *R. jostii* in the four-species microbial consortium in Wn medium. All experiments were performed at least in triplicate. Error bars indicate standard deviation. *p < 0.05, **p < 0.01 (one-sided *t*-test).

The capacity of single bacterial species with the microbial consortia to degrade PET was compared. Wild-type *B. subtilis*, *R. jostii*, and *P. putida* could not degrade PET (Figure 5A). The weight of the PET film decreased by 1.25 ± 0.14 mg after 7 days in the two-species microbial consortium, but by 2.80 ± 0.18 mg in the four-species consortium, doubling the degradation rate of the two-species consortium (Figure 5A). The four-species microbial consortium also successfully relieved the inhibitory effect of TPA. However, the concentration of TPA in the four-species microbial consortium increased on the 3rd day (Figure 5B). This may be because *R. jostii* preferentially used glucose in the early stage before metabolizing TPA.

The temperature, pH, and inoculation ratio of the four-species microbial consortium were further optimized. The most suitable temperature was 37°C (Supplementary Figure 5A), the pH had no obvious influence (Supplementary Figure 5B), and the optimal inoculation ratio of *Bs*_PETase: *Bs*_MHETase: *R. jostii*: *P. putida* was 3:1:10: 10^–5^ (Figures 5C,D). With these optimized parameters, the four-species microbial consortium reduced the weight of PET film by 7.9 ± 0.78 mg within 7 days (Figure 5D), a degradation rate of 23.2 ± 2.3%. Although the four-species microbial consortium could degrade EG, PET was not degraded as efficiently as it was by the three-species microbial consortium (31.2 ± 2.2%) because of the nutrient-poor medium. Both the three-species and four-species microbial consortia relieved the inhibitory effect of TPA. In terms of degradation efficiency, the three-species microbial consortium is superior, but the four-species microbial consortium is more environmentally friendly in terms of fully degrading the toxic products (TPA and EG) formed by breakdown of PET. It is therefore necessary to further optimize the relationship between the four bacteria to improve degradation efficiency.

## Discussion

Enzymatic degradation is currently considered an ideal method for PET degradation, and shows promise for large-scale PET waste degradation. Previous studies engineered and compared different signal peptides to enhance extracellular production of PETase in *E. coli* (Cui et al., 2021; Shi et al., 2021) and *B. subtilis* (Huang et al., 2018; Wang et al., 2020). These studies emphasized the importance of signal peptides in the secretion of heterologous protein. LipB from extracellular esterase was selected as the best signal peptide to secret PET-degrading enzymes, and two engineered *B. subtilis* strains that could secrete PETase and MHETase were generated. Most studies focused on the enzymatic degradation of PET *in vitro*, which needed to separate and purify the enzymes. Compared with degrading PET by purified enzymes, the degradation of PET by engineered *B. subtilis* in the fermentation system was studied, which is hopeful to realize large-scale industrial application.

In recent years, artificial microbial consortia that simulate natural microbial consortia to complete complex biological processes have become an important research direction of synthetic biology (Chen et al., 2020; Wang et al., 2020). Artificial microbial consortia introduced modularity to microbial metabolite by assigning different parts of the metabolic pathways to each member of the consortium (Zhou et al., 2015). The modular principle of artificial microbial consortia can distribute the complicated work to different microorganisms according to their own specific advantages, so as to complete the work that cannot be completed by a single microorganism. Only using a single microorganism to degrade PET and utilize the degradation products will increase the metabolic burden, making it difficult to achieve effective degradation. Constructing microbial consortia can reduce metabolic burden and promote biodegradation. A microbial consortium including three engineered *E. coli* was constructed to reduced metabolic burden to synthesize rosmarinic acid (Li Z. et al., 2019). Another consortium was constructed to produce short-chain fatty acids from lignocellulose, and it can reduce metabolic burden and perform multiple tasks (Shahab et al., 2020). These studies showed the potential to reduce metabolic burden by artificial consortia. A four-species microbial consortium consisting of *Bs*_PETase, *Bs*_MHETase, *R. jostii*, and *P. putida* was constructed to degrade PET. In this consortium, *B. subtilis* has the advantages of high secretion capacity, fast growth and lacking of an outer membrane, and it is regarded as a great chassis to secret heterologous proteins (Westers et al., 2004; van Dijl and Hecker, 2013). *B. subtilis* was chose as the model chassis and engineered it to secret PETase and MHETase, respectively. The two-species microbial consortium was considered as a PET-degrading module. Wild *R. jostii* and *P. putida* were reported to effectively use TPA and EG to support their growth, respectively (Choi et al., 2005; Wehrmann et al., 2017). The metabolic pathways of TPA and EG were considered as the TPA-degrading module and EG-degrading module. By combining the three modules to construct a four-species microbial consortium, PET can be degraded into monomers that could be absorbed and utilized by microorganisms capable of metabolizing them into carbon dioxide and water through the tricarboxylic acid cycle (Figure 2).

Artificial microbial consortia can relieve the inhibition of degradation products and improve the degradation rates. It has been demonstrated by a microbial consortium for corn fiber conversion to reduce the inhibition of hemicellulose hydrolysis products ethanol (Beri et al., 2020). It was previously shown that accumulation of MHET inhibited the function of PETase (Knott et al., 2020), likely explaining the increased degradation efficiency of the two-species consortium that was constructed (Figure 3A). Besides, the PET monomer TPA inhibited the degradation process. Therefore, *R. jostii* was added to the existing consortium to break down TPA, leading to a three-species microbial consortium with improved degradation efficiency. This three-species consortium could degrade more than 30% of PET within 7 days. By combining the PET-degrading module and the TPA-degrading module to construct a three-species microbial consortium, the inhibitory effect of TPA can be relieved, and the degradation efficiency can be improved (Figure 2).

Compared with pure culture, artificial microbial consortia have the advantages in stability and efficiency. The effects of environmental factors on the stability of the consortia were compared (Supplementary Figure 3). Results showed that the changes of temperatures and pH would not affect the stability of the two- and three-species microbial consortia (Supplementary Figure 3). These results showed the stability of the consortia that was constructed.

*Ideonella sakaiensis* 201-F6 could completely degrade low crystallinity PET film after 6 weeks at 30°C (Yoshida et al., 2016). However, *I. sakaiensis* 201-F6 is difficult to be genetically engineered due to its complex genetic background. This study constructed engineered *B. subtilis* to degrade PET, and the degradation products are completely converted into carbon dioxide and water. As far as degrading PET by using artificial microbial consortia, there are few studies on it. An artificial three-microbial consortium including *Exiguobacterium* sp., *Halomonas* sp., and *Ochrobactrum* sp. in a 1:1:1 ration was reported to degrade PET films, and PET films were fully degraded into small pieces after 2 weeks (Gao and Sun, 2021). In comparison with it, this study represents the first successful effort to degrade PET without any degradation products with an artificial microbial consortium, opening up new avenues for biodegradation of PET. The consortium described here not only degraded PET under ambient temperatures, but also addressed PETase inhibition by PET degradation products and improved the degradation efficiency. It reveals the potential of artificial microbial consortia in biodegradation of PET or other complex polymers. In the future, developing enhanced microorganism chassis and constructing artificial microbial consortia to degrade and convert PET to high-value chemicals is a promising method to realize the circular economy of PET waste.

## Conclusion

In this study, three artificial microbial consortia were constructed to degrade PET. Two *B. subtilis* was engineered to secrete PETase and MHETase, respectively. Wild *R. jostii* and *P. putida* were introduced to the microbial consortia to utilize PET monomers TPA and EG, respectively. A four-species microbial consortium including *Bs*_PETase, *Bs*_MHETase, *R. jostii*, and *P. putida* was constructed to directly degraded PET into monomers, and further converted them into carbon dioxide and water through the tricarboxylic acid cycle. The artificial microbial consortium successfully relieved the metabolic inhibition of TPA and EG, and effectively improved the degradation rate. The study provided novel ideas for the future biodegradation of PET or more other types of polymers by artificial microbial consortia.

## Data Availability Statement

The original contributions presented in the study are included in the article/Supplementary Material, further inquiries can be directed to the corresponding author/s.

## Author Contributions

XQ: conceptualization, methodology, validation, data curation, and writing—original draft. YM and HC: conceptualization, methodology, validation, and data curation. BL and YY: supervision. MD: conceptualization, writing—review and amp, editing, and supervision. All authors contributed to the article and approved the submitted version.

## Conflict of Interest

The authors declare that the research was conducted in the absence of any commercial or financial relationships that could be construed as a potential conflict of interest.

## Publisher’s Note

All claims expressed in this article are solely those of the authors and do not necessarily represent those of their affiliated organizations, or those of the publisher, the editors and the reviewers. Any product that may be evaluated in this article, or claim that may be made by its manufacturer, is not guaranteed or endorsed by the publisher.
