# Supplementary material for: Evaluation of PET Degradation Using Artificial Microbial Consortia
Source: Front Microbiol. 2021 Dec 23;12:778828. doi: 10.3389/fmicb.2021.778828 (PMC8733400; doi:10.3389/fmicb.2021.778828)
Supplement: Supplementary file 1 [file Data_Sheet_1.docx]

Supplementary Material

**Table S1** Strains used in this study

| **Strains** | **Description** | **Sources** | **Abbreviation** |
| --- | --- | --- | --- |
| *B. subtilis* 168 | Wild-type strain, trpC2 | BGSC^a^ | Bs |
| *B_S_*_PETase | *B. subtilis* 168; pHP13-*P_43_*-LipB-PETase | This study | B1 |
| *B_S_*_MHETase | *B. subtilis* 168; pHP13-*P_43_*-LipB-MHETase | This study | B2 |
| *P. putida* KT2440 | Wild-type strain | ATCC® 47054 | Pp |
| *R. jostii* RHA1 | Wild-type strain | Lindsay Eltis, Univ. British Columbia | Rj |
| *B_S_*_XynA-PETase | *B. subtilis* 168; pHP13-*P_43_*-XynA-PETase | This study |  |
| *B_S_*_BglS-PETase | *B. subtilis* 168; pHP13-*P_43_*-BglS-PETase | This study |  |
| *B_S_*_Csn-PETase | *B. subtilis* 168; pHP13-*P_43_*-Csn-PETase | This study |  |
| *B_S_*_SacB-PETase | *B. subtilis* 168; pHP13-*P_43_*-SacB-PETase | This study |  |
| *B_S_*_PelB-PETase | *B. subtilis* 168; pHP13-*P_43_*-PelB-PETase | This study |  |
| *B_S_*_AmyE-PETase | *B. subtilis* 168; pHP13-*P_43_*-AmyE-PETase | This study |  |
| *B_S_*_BglC-PETase | *B. subtilis* 168; pHP13-*P_43_*-BglC-PETase | This study |  |
| *B_S_*_YvpA-PETase | *B. subtilis* 168; pHP13-*P_43_*- YvpA-PETase | This study |  |

^a^ *Bacillus* Genetic Stock Center

**Table S2** Medium components

| **Medium** | **Components** |
| --- | --- |
| LB | 10 g/L tryptone, 5 g/L yeast extract, 10 g/L NaCl |
| YPD | 20 g/L glucose, 10 g/L yeast extract, 20 g/L peptone |
| SC | 20 g/L glucose, 6.7 g/L yeast nitrogen base without amino acids, and appropriate amino acids |
| W | 0.475 g/L ferrous sulfate heptahydrate, 0.118 g/L cobalt chloride hexahydrate, 1.11 g/L calcium chloride, 0.72 g/L zinc sulfate heptahydrate, 0.08 g/L copper sulfate, 0.03 g/L boric acid, 1.2 g/L glucose, 0.68 g/L potassium dihydrogen phosphate, 3.92 g/L disodium hydrogen phosphate, 0.4 g/L ammonium sulfate, 0.346 g/L ammonium chloride, 0.01 g/L magnesium chloride, 0.04 g/L magnesium sulfate heptahydrate |
| W_n_ | W Medium supplemented with 2 g/L potassium nitrate, 3 g/L potassium nitrate, 3 g/L glucose, 3 g/L sucrose |
| M9 | 6.78 g/L disodium hydrogen phosphate, 3 g/L potassium dihydrogen phosphate, 0.5 g/L sodium chloride, 1 g/L ammonium chloride, 0.493 g/L magnesium sulfate heptahydrate, 0.011 g/L calcium chloride, 4 g/L glucose |
| BM | 8.3 mg/L magnesium chloride hexahydrate, 0.535 g/L ammonium chloride, 0.0963 g/L ammonium nitrate, 1.35 g/L glutamic acid, 0.0198 g/L manganese chloride tetrahydrate, 0.106 g/L sodium sulfate, 0.148 g/L calcium chloride tetrahydrate, 15.7 g/L trimethylol aminomethane, 5 g/L glucose, 0.5 mg/L ferric chloride |
| TSM | 5 g/L glucose, 40 mg/L tryptophan, 20 mg/L magnesium sulfate, 440 mg/L potassium dihydrogen phosphate, 2 g/L sodium glutamate, 4 mg/L ferric chloride, 6.05 g/L tris. |
| SM | 5 g/L starch, 1 g/L magnesium sulfate heptahydrate, 0.05 g/L ferric chloride, 1 g/L potassium dihydrogen phosphate, 2.5 g/L disodium hydrogen phosphate, 0.5 g/L sodium chloride, 7.5 g/L ammonium sulfate, 1 g/L calcium chloride dihydrate, 0.025 g/L vitamin B1 |

**Table S3** Plasmids used in this study

| **Plasmids** | **Description** | **Sources** |
| --- | --- | --- |
| pHP13-*P_43_* | Cm^r^, Em^r^, *P_43_* promoter, *E. coli*-*B. subtilis* shuttle vector | Lab Stock |
| pHP13-*P_43_*-LipB-PETase | pHP13-*P_43_*; LipB-PETase | This study |
| pHP13-*P_43_*-LipB-MHETase | pHP13-*P_43_*; LipB-MHETase | This study |
| pHP13-*P_43_*-XynA-PETase | pHP13-*P_43_*; XynA-PETase | This study |
| pHP13-*P_43_*-BglS-PETase | pHP13-*P_43_*; BglS-PETase | This study |
| pHP13-*P_43_*-Csn-PETase | pHP13-*P_43_*; Csn-PETase | This study |
| pHP13-*P_43_*-SacB-PETase | pHP13-*P_43_*; SacB-PETase | This study |
| pHP13-*P_43_*-PelB-PETase | pHP13-*P_43_*; PelB-PETase | This study |
| pHP13-*P_43_*-AmyE-PETase | pHP13-*P_43_*; AmyE-PETase | This study |
| pHP13-*P_43_*-BglC-PETase | pHP13-*P_43_*; BglC-PETase | This study |
| pHP13-*P_43_*-YvpA-PETase | pHP13-*P_43_*; YvpA-PETase | This study |

**Table S4** DNA sequences of PETase and MHETase

| **Genes** | **DNA sequences** |
| --- | --- |
| PETase | CAGACAAATCCGTATGCAAGAGGCCCGAATCCGACAGCAGCATCACTGGAAGCATCAGCAGGCCCGTTTACAGTTCGCAGCTTCACAGTTTCAAGACCGTCAGGCTATGGCGCAGGCACAGTTTATTATCCGACAAATGCAGGCGGAACAGTTGGCGCAATTGCAATTGTTCCGGGCTATACAGCAAGACAAAGCAGCATTAAATGGTGGGGCCCGAGATTAGCATCACATGGCTTCGTTGTTATTACGATCGACACAAATTCAACGCTGGATCAACCGTCATCAAGATCATCACAACAAATGGCAGCACTGAGACAAGTTGCATCACTGAATGGCACATCATCATCACCGATCTATGGCAAAGTTGACACAGCAAGAATGGGCGTTATGGGCTGGTCAATGGGAGGAGGCGGCTCACTTATTTCAGCAGCAAATAACCCGTCACTGAAAGCAGCAGCACCTCAAGCACCGTGGGATTCATCAACAAACTTCTCATCAGTTACAGTGCCGACACTGATTTTTGCATGCGAGAACGATTCAATCGCACCGGTTAATTCATCAGCACTGCCGATTTATGATTCAATGAGCCGCAACGCAAAACAATTCCTGGAAATCAATGGCGGCTCACATTCATGCGCAAATTCAGGCAATTCAAACCAAGCACTGATTGGCAAAAAAGGCGTTGCATGGATGAAAAGATTCATGGACAATGATACGCGCTACTCAACATTTGCATGCGAAAACCCGAACTCAACAAGAGTGAGCGATTTTCGCACAGCAAATTGCTCATAAGAATTC |
| MHETase | GATCCATGAAATTTGTAAAAAGAAGGATCATTGCACTTGTAACAATTTTGATGCTGTCTGTTACATCGCTGTTTGCGTTGCAGCCGTCAGCAAAAGCCGGCGGAGGATCAACACCTTTACCGCTGCCTCAACAACAACCGCCGCAACAAGAACCTCCTCCGCCTCCTGTTCCTCTGGCATCAAGAGCAGCATGCGAAGCACTGAAAGATGGCAATGGCGATATGGTTTGGCCGAATGCAGCAACAGTTGTTGAAGTTGCAGCATGGAGAGATGCAGCACCGGCAACAGCATCAGCAGCAGCACTGCCGGAACATTGCGAAGTTTCAGGCGCAATTGCAAAAAGAACAGGCATTGATGGCTATCCGTACGAGATTAAGTTTAGACTGAGAATGCCGGCAGAATGGAATGGCCGCTTTTTTATGGAAGGCGGCTCAGGCACAAATGGCTCACTGTCAGCAGCAACAGGCTCAATTGGCGGCGGCCAAATTGCATCAGCACTGTCAAGAAACTTTGCGACGATTGCAACAGATGGCGGCCATGATAATGCAGTTAACGATAATCCGGATGCACTGGGCACAGTTGCATTTGGCCTGGACCCGCAAGCAAGACTGGATATGGGCTATAACAGCTATGACCAGGTTACACAAGCAGGCAAAGCAGCAGTTGCAAGATTTTATGGCAGAGCAGCAGATAAGTCATACTTTATTGGCTGCTCAGAAGGCGGAAGAGAAGGCATGATGCTGTCACAAAGATTTCCGTCACATTACGATGGCATTGTTGCAGGCGCACCGGGATATCAACTTCCGAAAGCAGGCATTTCAGGCGCATGGACAACACAATCACTGGCACCGGCAGCAGTTGGCTTAGATGCACAAGGCGTTCCGCTGATTAATAAGTCATTCAGCGACGCAGACCTGCATCTGCTGTCACAAGCAATTCTGGGCACATGCGATGCACTTGATGGCCTGGCAGATGGCATTGTGGATAATTATAGAGCATGCCAGGCAGCATTTGATCCGGCAACAGCAGCAAATCCGGCAAATGGCCAAGCACTGCAATGCGTTGGCGCAAAAACAGCAGATTGCCTGTCACCGGTTCAAGTTACGGCAATCAAAAGAGCAATGGCAGGCCCGGTTAATTCAGCAGGCACACCGCTGTATAATAGATGGGCATGGGATGCAGGAATGTCAGGCCTGTCAGGCACAACATATAATCAAGGCTGGAGATCATGGTGGCTGGGCTCATTTAATTCATCAGCGAACAATGCACAAAGAGTGTCAGGCTTTTCAGCAAGATCATGGCTGGTTGATTTTGCAACACCGCCGGAACCTATGCCGATGACACAAGTTGCAGCGAGAATGATGAAGTTCGATTTCGATATCGACCCGCTGAAAATTTGGGCAACAAGCGGCCAATTTACACAATCAAGCATGGATTGGCATGGCGCAACATCAACGGATCTGGCAGCATTTAGAGATAGAGGCGGCAAAATGATTCTGTATCACGGCATGTCAGATGCAGCATTTTCAGCACTGGATACAGCAGATTACTATGAAAGACTGGGCGCAGCAATGCCTGGAGCAGCAGGATTTGCAAGACTGTTTCTGGTTCCGGGCATGAATCATTGTTCAGGCGGCCCTGGCACAGATAGATTTGATATGCTGACACCGCTGGTTGCATGGGTTGAAAGAGGCGAAGCACCGGATCAAATTTCAGCATGGTCAGGCACACCGGGCTATTTTGGCGTTGCAGCAAGAACAAGACCGCTGTGCCCGTATCCTCAAATTGCACGCTATAAAGGCTCAGGCGACATTAATACGGAAGCGAACTTTGCATGCGCAGCACCTCCGTAAGAATT |

**Table S5** Amino acid sequences of signal peptides

| **Signals** | **Amino acid sequences** | **Source** |
| --- | --- | --- |
| XynA | MFKFKKNFLVGLSAALMSISLFSATASA | Endo-1,4-β-xylanase A |
| BglS | MPYLKRVLLLLVTGLFMSLFAVTATASA | β-glucanase |
| Csn | MKISMQKADFWKKAAISLLVFTMFFTLMMSETVFA | Chitosanase |
| SacB | MNIKKFAKQATVLTFTTALLAGGATQAFA | Fructansucrase |
| PelB | MKRLCLWFTVFSLFLVLLPGKALG | Pectin lyase |
| BglC | MKRSISIFITCLLITLLTMGGMIASPASA | Aryl phosphate-β-D-glucosidase |
| YvpA | MKKIVSILFMFGLVMGFSQFQPSTVFA | Pectin lyase |
| LipB | LAKKDEHLRKPEWLKIKLNTNENYTGLKKLMREN | Extracellular esterase |
| AmyE | MFAKRFKTSLLPLFAGFLLLFHLVLAGPAAASA | α-amylase |

**Table S6** Primes used in this study

| **Primers** | **Sequence (5′-3′)** | **Application** |
| --- | --- | --- |
| LipB-F | GGATCCATGAAATTTGTAAAAAGAAGGATCATTG | Forward primer for the amplification of  LipB-PETase |
| Lip-R1 | CTTGCATACGGATTTGTCTGGGCTTTTGCTGACGGC | Reverse primer for the amplification of  LipB |
| Lip-PETase-F | TGCAGCCGTCAGCAAAAGCCCAGACAAATCCGTATGCAAGA | Forward primer for the amplification of  LipB-PETase |
| PETase-R | GAATTCTTATGAGCAATTTGCTGTGC | Reverse primer for the amplification of  PETase |
| Lip-R2 | GTCGAACCCCCACCTGCGCAGGCTTTTGCTGACGGCT | Forward primer for the amplification of  LipB-MHETase |
| MHET-F | TGCAGCCGTCAGCAAAAGCCTGCGCAGGTGGGGGTTCGAC | Forward primer for the amplification of  MHETase |
| MHET-R | GAATTCTTACGGAGGGGCTGCACAGGCAAAAT | Reverse primer for the amplification of  MHETase |
| XynA-F | GGATCCATGTTTAAATTTAAAAAAAACTTTCTGGTCG | Forward primer for the amplification of  XynA |
| XynA-R | CTTGCATACGGATTTGTCTGGGCGCTTGCTGTCGC | Reverse primer for the amplification of  XynA |
| XynA-PETase-F | TTTCGGCGACAGCAAGCGCCCAGACAAATCCGTATGCAAGA | Forward primer for the amplification of  XynA-PETase |
| BglS-F | GGATCCATGCCGTATCTGAAACGG | Forward primer for the amplification of  BglS |
| BglS-R | CTTGCATACGGATTTGTCTGGGCGCTCGCTGTTGC | Reverse primer for the amplification of  BglS |
| BglS-PETase-F | TTACCGCAACAGCGAGCGCCCAGACAAATCCGTATGCAAGA | Forward primer for the amplification of  BglS-PETase |
| Csn-F | GGATCCATGAAAATCTCTATGCAAAAGGC | Forward primer for the amplification of  Csn |
| Csn-R | CTTGCATACGGATTTGTCTGGGCAAACACTGTTTCCGA | Reverse primer for the amplification of  Csn |
| Csn-PETase-F | TGTCGGAAACAGTGTTTGCCCAGACAAATCCGTATGCAAGA | Forward primer for the amplification of  Csn-PETase |
| SacB-F | GGATCCATGAATATCAAAAAATTTGCCAAACAG | Forward primer for the amplification of  SacB |
| SacB-R | ATTCGGGCCTCTTGCATACGGATTTGTCTGCGCAAATGCCTGTGTG | Reverse primer for the amplification of  SacB |
| SacB-PETase-F | GAGCCACACAGGCATTTGCGCAGACAAATCCGTATGCAAGA | Forward primer for the amplification of  SacB-PETase |
| PelB-F | GGATCCATGAAACGGCTGTGTTTATGG | Forward primer for the amplification of  PelB |
| PelB-R | CTTGCATACGGATTTGTCTGGCCTAACGCTTTGCCAG | Reverse primer for the amplification of  PelB |
| PelB-PETase-F | TGCCTGGCAAAGCGTTAGGCCAGACAAATCCGTATGCAAGA | Forward primer for the amplification of  PelB-PETase |
| AmyE-F | GGATCCATGTTTGCAAAACGCTTTAAAACA | Forward primer for the amplification of  AmyE |
| AmyE-R | CTTGCATACGGATTTGTCTGAGCGCTCGCGGC | Reverse primer for the amplification of  AmyE |
| AmyE-PETase-F | GCCCGGCAGCCGCGAGCGCTCAGACAAATCCGTATGCAAGA | Forward primer for the amplification of  AmyE-PETase |
| BglC-F | GGATCCATGAAAAGATCTATTAGCATTTTTATCAC | Forward primer for the amplification of  BglC |
| BglC-R | CTTGCATACGGATTTGTCTGCGCGCTTGCCGGTGAG | Reverse primer for the amplification of  BglC |
| BglC-PETase-F | TTGCCTCACCGGCAAGCGCGCAGACAAATCCGTATGCAAGA | Forward primer for the amplification of  BglC-PETase |
| YvpA-F | GGATCCATGAAAAAAATTGTCAGCATCTTATT | Forward primer for the amplification of  YvpA |
| YvpA-R | CTTGCATACGGATTTGTCTGTGCAAACACTGTTGAAGGTT | Reverse primer for the amplification of  YvpA |
| YvpA-PETase-F | AACCTTCAACAGTGTTTGCACAGACAAATCCGTATGCAAGA | Forward primer for the amplification of  YvpA-PETase |

^a^ Underlining represents restriction sites used for cloning.

**A B**


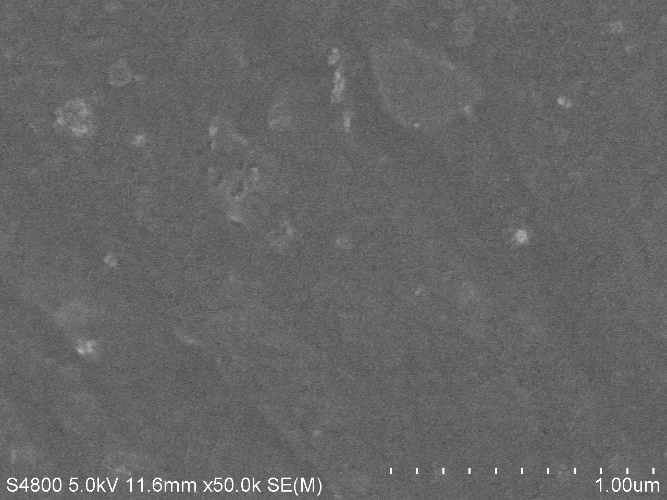

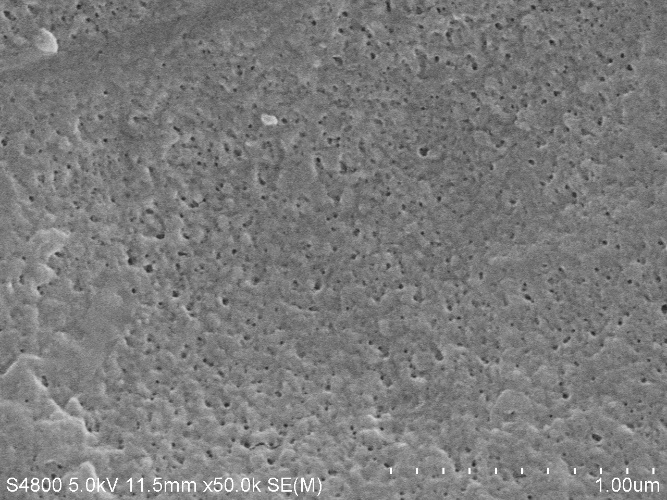


**Fig. S1 Scanning electron microscope (SEM) image of PET films’ surface.** (A) SEM image of PET film surface cultured with wild *B. subtilis*. (magnification: 50k) (B) SEM image of PET film surface cultured with *Bs*_PETase for 7 days. (magnification: 50k)

**Fig. S2** **Degradation rates of BHET at different initial concentrations.**

**A B**

**C D**

**Fig. S3 Temperature and pH optimization of the two- and three species microbial consortium.** (A)Time required for the two-species microbial consortium to completely degrade 2 g/L BHET at different temperatures. (B) Time required for the two-species microbial consortium to completely degrade 2 g/L BHET at different pH levels. (C)Time required for the three-species microbial consortium to completely degrade 2 g/L BHET at different temperatures. (D) Time required for the three-species microbial consortium to completely degrade 2 g/L BHET at different pH levels.

| **A** | **B** |
| --- | --- |
|  |  |
| **C** | **D** |
|  |  |
| **E** |  |
|  |  |

**Fig. S4. Selection and optimization of microbial consortia.** (A) Concentration of EG during incubation with *P. putida* (dark blue circles) and OD_600_ of *P. putida* grown in LB medium with (orange triangles) or without (light blue squares) 2g/L EG. (B) Comparison of microbial consortium growth in different culture media. (C) Growth of the individual bacterial species with different carbon sources. (D) Growth of the individual bacterial species with different nitrogen sources. (E) Concentrations of BHET, TPA, and EG during incubation with the four-species microbial consortium. All experiments were performed at least in triplicate. Error bars indicate standard deviation.

| **A** | **B** |
| --- | --- |
|  |  |

**Fig. S5. Optimization of growth temperature and growth medium pH for the four-species microbial consortia.** (A) Optimization of growth temperature for the four-species microbial consortia in Wn medium. (B) Optimization of growth medium pH for the four-species microbial consortia in Wn medium. All experiments were performed at least in triplicate. Error bars indicate standard deviation. **p* < 0.05, ***p* < 0.01, ****p* < 0.001 (one-sided t-test).
